# Exploring tumor-derived exosomes as promising antigenic platforms: novel vaccine and immunotherapy strategies for “cold” colorectal cancer

**DOI:** 10.3389/fimmu.2026.1962696

**Published:** 2026-09-29

**Authors:** Xiaoting Liu, Xiaoyan Wang

**Affiliations:** 1 Huzhou Central Hospital, Fifth School of Clinical Medicine of Zhejiang Chinese Medical University, Huzhou, Zhejiang, China; 2 Huzhou Central Hospital, Affiliated Central Hospital of Huzhou University, Huzhou, Zhejiang, China

**Keywords:** cancer vaccines, colorectal cancer, exosome engineering, immunotherapy, tumor microenvironment, tumor-derived exosomes

## Abstract

Colorectal cancer (CRC) remains a formidable clinical challenge, largely due to the prevalence of microsatellite-stable tumors characterized by an immunologically “cold” tumor microenvironment (TME) that is resistant to immune checkpoint inhibitors (ICIs). Tumor-derived exosomes (TEXs) play a paradoxical dual role in this landscape. Native TEXs actively orchestrate immune evasion by delivering immunosuppressive molecules, such as programmed cell death 1 ligand 1 (PD-L1) and transforming growth factor-beta (TGF-β), which impair cytotoxic T-cell function and promote the expansion of regulatory T cells and M2 macrophages. Conversely, TEXs possess inherent biocompatibility and naturally harbor a broad repertoire of tumor-associated antigens (TAAs) and major histocompatibility complex (MHC) molecules, positioning them as potential cell-free platforms for cancer vaccination. Current evidence demonstrates that native TEXs establish their immunosuppressive niche primarily through the systemic PD-1/PD-L1 axis and microRNA-driven macrophage polarization. However, preclinical studies indicate that exosome engineering strategies—such as masking surface PD-L1 or loading vesicles with immune adjuvants, siRNAs, or CRISPR components—can effectively neutralize these suppressive traits and amplify antigen presentation. While clinical data in CRC remain limited, engineered TEXs exhibit strong preclinical promise for synergizing with ICIs to reprogram cold CRC into immune-responsive “hot” tumors. Advancing these early-stage therapies from the bench to the clinic will require overcoming significant translational hurdles, particularly regarding standardized isolation protocols and scalable manufacturing.

## 1 Introduction

Colorectal cancer (CRC) remains a prevalent and lethal malignancy (1, 2), with advanced stages often yielding limited responses to conventional therapies (3, 4). Although immune checkpoint inhibitors (ICIs) have transformed oncology, their efficacy in CRC is primarily restricted to the microsatellite instability-high (MSI-H) subtype, which constitutes only approximately 15% of cases (5, 6). The majority of patients harbor microsatellite-stable (MSS) tumors, which are immunologically “cold” and resistant to ICI monotherapy (7, 8). These cold tumors are characterized by low T-cell infiltration and a profoundly immunosuppressive tumor microenvironment (TME) dominated by regulatory T cells (Tregs), M2-polarized tumor-associated macrophages (TAMs), and myeloid-derived suppressor cells (MDSCs), which collectively impede cytotoxic T lymphocyte (CTL) activity (5, 7, 9, 10). Overcoming this immunological barrier to convert cold CRC into ICI-responsive “hot” tumors represents a formidable clinical challenge.

Tumor-derived exosomes (TEXs)—nanoscale (30–150 nm) extracellular vesicles carrying diverse biomolecular cargo from their parental cells (11, 12)—have emerged as key modulators of tumor immunobiology and promising therapeutic platforms (13, 14). In CRC, TEXs exhibit a profoundly paradoxical dual role, creating a constant biological tension between immune evasion and immune activation (15, 16). On one hand, they act as potent effectors of immune suppression by delivering molecules (e.g., PD-L1, TGF-β, and FasL) that actively paralyze T-cell function and drive M2/Treg polarization, thereby enforcing the cold TME (17–20). On the other hand, unlike synthetic nanocarriers, native TEXs possess inherent biocompatibility and low immunogenicity (21, 22). They naturally harbor a broad repertoire of tumor-associated antigens (TAAs), including carcinoembryonic antigen (CEA), alongside major histocompatibility complex (MHC) molecules, presenting a theoretically ideal cell-free platform for cross-priming (4, 23–25). Crucially, a critical analysis of the literature reveals that in the natural TME, the immunosuppressive signaling of TEXs evolutionarily outcompetes their antigen-presenting potential, resulting in a net outcome of profound immune tolerance. Therefore, harnessing TEXs for therapy is not simply about utilizing their natural state; rather, it requires extensive biological engineering to forcefully tip this delicate stoichiometric balance—dismantling their suppressive traits while amplifying their immunogenic cargo to unlock their true potential as personalized immunotherapies (19, 20).

This review explores the paradoxical roles of TEXs in cold CRC. We discuss the mechanisms by which TEXs orchestrate TME immunosuppression, evaluate their potential as natural antigen platforms for cancer vaccination, and review state-of-the-art engineering strategies designed to augment their immunotherapeutic efficacy. Finally, we address the translational challenges and future directions for advancing TEX-based therapies from bench to bedside.

## 2 Tumor-derived exosomes as mediators of immunosuppression in the “cold” colorectal cancer microenvironment

The immunosuppressive TME of cold CRC is shaped by complex intercellular communication networks in which TEXs play a central role (17, 18). TEXs carry a molecular cargo that reflects the malignant phenotype of their parental cells and actively reprogram recipient immune cells to promote tumor immune evasion (26, 27). Understanding these mechanisms is critical for developing strategies to overcome immunosuppression and enhance immunotherapy responses.

### 2.1 Direct immune checkpoint and apoptotic signaling

A particularly well-characterized mechanism of TEX-mediated immunosuppression involves the PD-1/PD-L1 axis. TEXs carry functional PD-L1 on their surface, which can bind to PD-1 receptors on T cells and suppress their effector functions systemically (28–30). In CRC, exosomal PD-L1 has been detected in patient plasma and correlates with disease progression and poor prognosis (31, 32). Importantly, exosomal PD-L1 can mediate resistance to anti-PD-1/PD-L1 immunotherapy by acting as a decoy that neutralizes therapeutic antibodies or by delivering inhibitory signals to T cells in the tumor-draining lymph nodes (28, 33). The suppression of exosomal PD-L1 secretion has been shown to induce systemic antitumor immunity and long-term memory in preclinical models, highlighting its therapeutic potential (33). Furthermore, CRC-derived exosomes carry the long non-coding RNA (lncRNA) HOTAIR, which induces PD-L1 expression on B cells, generating regulatory B cells that suppress CD8+ T cell activity (34).

Beyond checkpoint inhibition, TEXs directly suppress immune effector cells through apoptotic signaling. They induce apoptosis of activated CD8+ T cells through FasL and TRAIL signaling, and promote the differentiation of naive T cells into Tregs, shifting the balance toward immunosuppression (35, 36). TEXs also inhibit natural killer (NK) cell activation by downregulating activating receptors such as NKG2D and by transferring TGF-β, which suppresses NK cell cytotoxicity (37, 38). These direct receptor-ligand interactions represent a foundational pillar of the cold CRC phenotype. Consequently, therapeutic strategies that strip native TEXs of surface PD-L1—either through genetic ablation (e.g., CRISPR/Cas9) or surface masking—are critical preliminary steps in transforming TEXs from immunosuppressive mediators into viable immunogenic vaccine platforms.

### 2.2 Myeloid cell reprogramming and antigen presentation deficits

TEXs heavily modulate myeloid cell populations within the TME to establish an antigen-presentation deficit. They impair dendritic cell (DC) differentiation and maturation, thereby compromising antigen presentation and T cell priming (39, 40). CRC-derived exosomes have been shown to inhibit monocyte differentiation into functional DCs and instead promote the generation of immunosuppressive myeloid cells, including the expansion of MDSCs (41).

Macrophage polarization is another key target of TEX-mediated immunomodulation in CRC. TAMs in cold CRC predominantly exhibit an M2-like, immunosuppressive phenotype that supports tumor growth and metastasis (9, 42). CRC-derived exosomes deliver specific microRNAs (miRNAs), including miR-21-5p, miR-200a, and miR-934, which downregulate PTEN expression and activate the PI3K/AKT signaling pathway in macrophages, driving their polarization toward the M2 phenotype (9, 43). This exosome-mediated crosstalk establishes a positive feedback loop: M2 macrophages secrete cytokines such as CXCL13, which further promotes tumor cell survival and exosome production (43). Because CRC primarily drains through the portal venous system, the liver serves as the dominant site for metastasis, making the hepatic pre-metastatic niche a highly CRC-specific phenomenon (44, 45). Unlike generic pan-cancer models, CRC-derived exosomes explicitly exploit this anatomical route to continuously educate liver-resident Kupffer cells and hepatic stellate cells. A prominent example is the transfer of exosomal miR-934, which uniquely induces M2 polarization in Kupffer cells and triggers the secretion of inflammatory cytokines, thereby cultivating a fibrotic, immunosuppressive “soil” specifically tailored for CRC cell colonization and outgrowth in the liver (43, 45). By simultaneously stalling DC maturation and driving M2 macrophage and MDSC expansion, TEX-delivered non-coding RNAs form a profound biochemical barrier that traps CRC in an immunologically barren state. This underscores the therapeutic imperative of engineered exosomes loaded with targeted siRNAs or microRNA antagonists, which are specifically designed to intercept these myeloid-reprogramming signals and restore endogenous antigen presentation.

### 2.3 Metabolic reprogramming and hypoxia-driven suppression

Hypoxia, a hallmark of the solid TME, profoundly influences TEX composition and function (46, 47). Under hypoxic conditions, tumor cells increase exosome secretion and alter their cargo to include hypoxia-inducible factor 1α (HIF-1α)-regulated molecules that promote angiogenesis, metastasis, and immune evasion (47, 48). Hypoxic TEXs from CRC cells have been shown to induce M2 macrophage polarization, enhance Treg recruitment, and suppress CTL activity, thereby reinforcing the cold tumor phenotype (46, 49). The hypoxia-TEX axis represents a critical pathway linking the metabolic stress of the TME to systemic immunosuppression.

The immunosuppressive cargo of TEXs extends beyond hypoxia-related proteins to include lipids, non-coding RNAs, and metabolites that drive metabolic reprogramming (50, 51). Exosomal circRNAs and lncRNAs have been implicated in enhancing MDSC suppressive activity (51). Crucially, TEXs carry metabolic enzymes such as CD39 and CD73 that generate adenosine, a potent immunosuppressive metabolite that fundamentally inhibits T cell and NK cell function (52, 53). Importantly, the CRC TME is uniquely segregated from other solid tumors by its constant exposure to the gut microbiome. Emerging evidence indicates a profound, CRC-specific cross-talk where intestinal microbiota and their derived metabolites actively modulate the biogenesis and cargo sorting of CRC TEXs. This microbiota-TEX axis forms a dual modulatory system that exacerbates local immune exclusion, specifically reinforcing the therapy-resistant architecture of MSS “cold” tumors (45, 54). The hypoxia-driven metabolic shifts, particularly the exosomal CD39/CD73 adenosinergic pathway, lock the CRC microenvironment into a persistent state of metabolic exhaustion for effector immune cells. Translating this mechanistic insight into therapy highlights the potential of engineering smart exo-nanomaterials capable of delivering metabolic modulators or hypoxia-responsive elements to dismantle this hostile metabolic niche and sensitize the TME to checkpoint blockade.

Importantly, an in-depth assessment of these immunosuppressive mechanisms uncovers the fundamental paradox of TEX biology: how can a vesicle that so potently orchestrates immune evasion simultaneously serve as a vaccine platform? The answer lies in the stoichiometry of their cargo and the biological context of their presentation. In their native, steady-state secretion within the TME, TEXs operate as a systemic “tolerogenic swarm”. The sheer abundance of suppressive ligands overwhelmingly eclipses their endogenous antigen-presenting capacity, establishing a net-negative immune threshold that actively silences T cells and promotes M2/MDSC expansion. However, this immunosuppressive dominance is not an irreversible structural flaw, but rather an acquired malignant phenotype. By chemically or genetically stripping these suppressive factors, the biological “tug-of-war” can be decisively tipped. Once the suppressive overlay is neutralized, the underlying architecture of the TEX—its rich repertoire of tumor-associated antigens and endogenous adjuvants—is completely unmasked. This critical mechanistic insight shifts the therapeutic paradigm: strategies must evolve beyond simply inhibiting TEX biogenesis to actively coopting their antigenic potential (55, 56). The subsequent sections critically examine how this biological reprogramming is achieved, evaluating how engineering strategies successfully invert the native suppressive stoichiometry to unleash TEXs as robust immunotherapeutic platforms.

## 3 Exploiting tumor-derived exosomes as natural antigen platforms for cancer vaccination

Despite their immunosuppressive functions, TEXs possess intrinsic properties that make them attractive platforms for cancer vaccination. This section evaluates the hierarchical evidence supporting TEXs as vaccine platforms, ranging from biological rationale to clinical applications and engineering enhancements.

### 3.1 Biological rationale of TEX-based vaccines

TEXs carry a comprehensive repertoire of tumor antigens, including TAAs, neoantigens, and MHC-peptide complexes, enabling direct presentation to immune cells and cross-priming of T cell responses (15, 24, 25). This antigenic diversity is a significant advantage over synthetic vaccines that target only a limited number of defined antigens, as TEXs can potentially elicit broader and more robust antitumor immune responses. The immunogenicity of TEXs depends heavily on their native cargo composition. Under appropriate conditions, they can stimulate both innate and adaptive immune responses. They carry heat shock proteins (HSPs, e.g., HSP70, HSP90) that act as endogenous adjuvants, activating DCs and promoting antigen cross-presentation (57, 58). Additionally, TEXs contain damage-associated molecular patterns (DAMPs) that can trigger pattern recognition receptors on innate immune cells, enhancing the immunogenicity of the vaccine (10). These properties suggest that TEXs can function as self-adjuvanting antigen delivery systems, potentially eliminating the need for exogenous adjuvants.

### 3.2 Preclinical proof-of-concept and clinical evidence

The concept of using TEXs as cancer vaccines dates back to early preclinical studies demonstrating that TEXs could transfer antigens to DCs and induce potent CD8+ T cell-dependent antitumor effects in mouse models (24). Subsequent work confirmed that TEXs contain immunogenic tumor antigens that can be processed and presented by DCs to prime naive T cells, leading to robust tumor rejection (15, 25). In CRC specifically, TEXs have been rigorously investigated as vaccine platforms in both preclinical and early clinical settings. Ascites-derived exosomes from CRC patients were shown to contain CEA and other TAAs, and their administration in combination with GM-CSF in a phase I clinical trial induced tumor-specific CTL responses with acceptable safety profiles (23). While this proof-of-concept study successfully demonstrated the safety of TEX-based vaccination, it is crucial to acknowledge the negative clinical data: the trial failed to demonstrate meaningful objective response rate (ORR) or prolonged progression-free survival (PFS) in advanced CRC patients (23). This suboptimal clinical outcome provides a sobering translational lesson, confirming that native TEXs, without extensive engineering or combinatorial adjuvants, are insufficiently potent to overcome established immune tolerance in human trials.

### 3.3 Strategies to enhance vaccine efficacy

To overcome limited clinical efficacy, the immunogenicity of TEX-based vaccines can be significantly enhanced through various engineering and combinatorial strategies. One approach involves engineering tumor cells to overexpress HSP70 before exosome isolation, yielding TEXs with enhanced DC activation capacity and improved antitumor efficacy (58, 59). Similarly, TEXs can be externally loaded with Toll-like receptor (TLR) agonists or cytokines to create more potent vaccine formulations (60, 61). The antigenic content of TEXs can also be artificially enriched. Tumor cells treated with immunogenic cell death (ICD) inducers, such as radiotherapy, secrete TEXs containing elevated levels of DAMPs. For instance, radiation-induced TEXs have been shown to transfer IFN-stimulatory dsDNA to DCs, activating the cGAS/STING pathway and massively enhancing antitumor immunity (10, 62).

Furthermore, the recent success of mRNA vaccines has inspired the development of exosome-based nucleic acid vaccines. Engineered exosomes can be loaded with mRNA encoding tumor antigens or immunostimulatory molecules, combining the advantages of exosomal delivery with the flexibility of nucleic acid-based vaccination (63, 64). Finally, combining TEX vaccination with immune checkpoint blockade is critical for overcoming the immunosuppressive TME. In mouse models of CRC, neoantigen-painted exosome vaccines combined with anti-PD-1 therapy achieved complete tumor regression and established durable immunological memory (65), representing a highly promising strategy for converting cold CRC to a responsive phenotype.

### 3.4 Translational limitations and alternative platforms

The use of TEXs as vaccines offers several practical advantages over conventional DC-based vaccination: they are cell-free, resistant to freeze-thaw cycles, can be standardized easily (22, 66), and can be administered systemically without the risk of inducing cellular anergy (16). However, significant translational limitations remain. The inherent immunosuppressive cargo of native TEXs poses a major challenge. Notably, a rigorous examination of the literature highlights several explicitly negative preclinical outcomes where unmodified TEX vaccines failed to prime T cells and instead inadvertently accelerated tumor growth by inducing systemic anergy (16, 67). These failed experimental attempts powerfully illustrate the profound biological risk of utilizing TEXs without neutralizing their native tolerogenic cargo. To prevent such negative outcomes, contemporary approaches mandate chemical or genetic modifications to remove or mask immunosuppressive surface proteins, such as PD-L1, while preserving antigenic molecules (19, 20), or employ inhibitors of exosome biogenesis to therapeutically alter their cargo sorting (14, 55).

Another limitation lies in the selection of appropriate antigen sources. While TEXs from autologous tumor cells provide a personalized neoantigen repertoire, this approach is logistically challenging (15, 65). Allogeneic TEXs from established tumor cell lines offer a more scalable alternative but may lack sufficient personalized antigenic coverage (61, 65). Due to these hurdles, dendritic cell-derived exosomes (DEXs) represent a viable alternative platform. DEXs carry MHC-peptide complexes and costimulatory molecules enabling direct T cell activation without the immunosuppressive cargo characteristic of TEXs (66, 68). Preclinical studies and early clinical trials of DEXs have demonstrated strong safety and immunogenicity (66, 69), though their production requires complex DC culture and antigen loading, adding significant cost compared to TEX-based approaches.

## 4 Engineering exosomes to augment immunotherapy responses in cold colorectal cancer

The engineering of exosomes has emerged as a powerful strategy to enhance their therapeutic potential and address the limitations of native TEXs for cancer immunotherapy (19, 20, 70). Various engineering approaches have been developed to improve exosome targeting, cargo loading, immunogenicity, and therapeutic efficacy, offering new possibilities for converting cold CRC to an immunotherapy-responsive phenotype (**Figure 1**). To better visualize the translational landscape, we have summarized the key representative studies and essential quality control parameters in a comprehensive master table (**Table 1**). As illustrated, while there is a robust and expanding body of preclinical evidence demonstrating the efficacy of engineered TEXs and combinatorial strategies, actual clinical evidence in CRC remains limited, underscoring the critical need for continued translational research.

**Figure 1:**
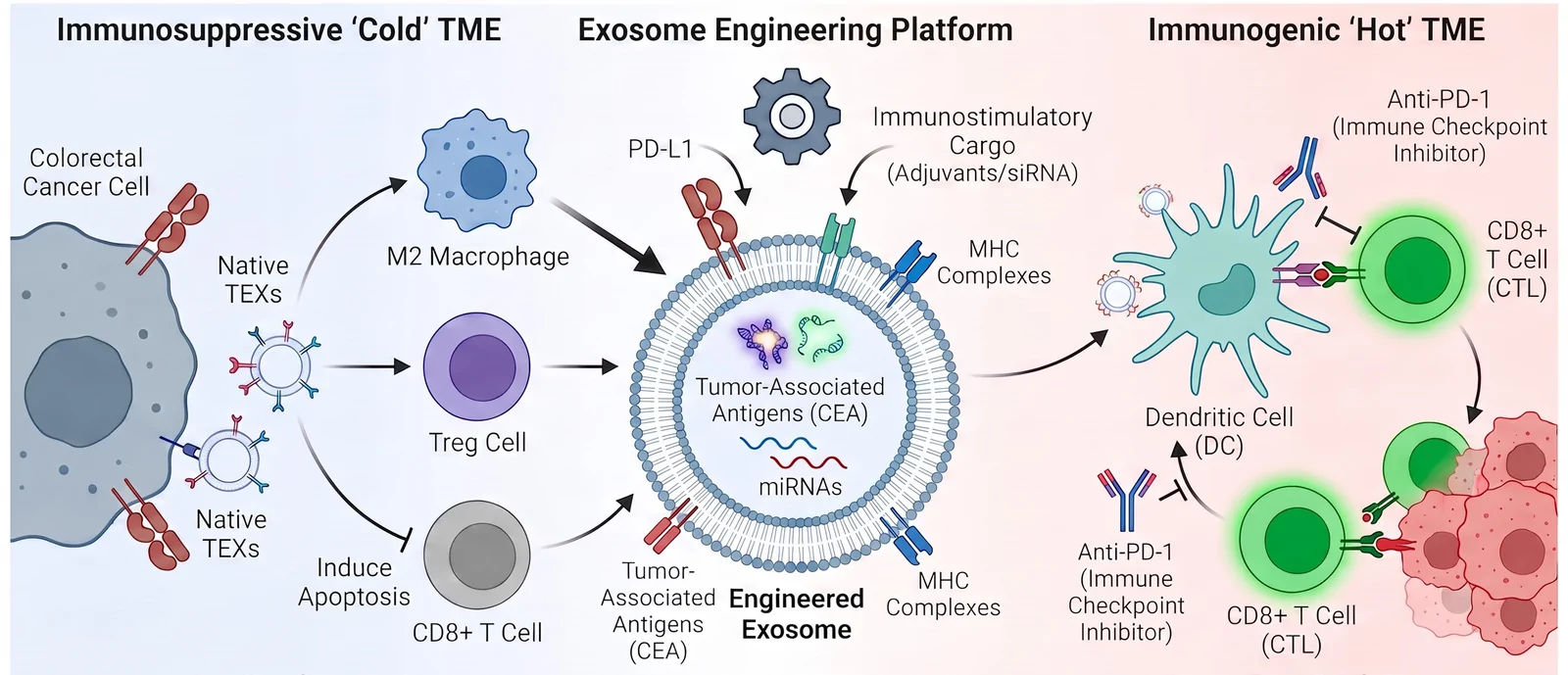
The paradoxical role of tumor-derived exosomes (TEXs) in colorectal cancer and the exosome engineering platform for remodeling the tumor microenvironment (TME). (Left) In the immunosuppressive “cold” TME, native TEXs secreted by colorectal cancer cells deliver inhibitory molecules (e.g., PD-L1 and FasL) that induce M2 macrophage polarization, promote regulatory T cell (Treg) expansion, and trigger apoptosis or functional exhaustion of CD8+ T cells, culminating in low T-cell infiltration and immune evasion. (Middle) The exosome engineering platform reprograms these vesicles by masking/removing surface PD-L1 and loading immunostimulatory cargos (adjuvants, siRNAs). The engineered exosomes are enriched with tumor-associated antigens (such as CEA) and major histocompatibility complex (MHC) molecules. (Right) In the immunogenic “hot” TME, engineered exosomes function as robust vaccines. They effectively present antigens to dendritic cells (DCs) and synergize with anti-PD-1 therapies to overcome immune checkpoints. This leads to the robust activation and infiltration of cytotoxic T lymphocytes (CTLs), ultimately driving tumor rejection.

**Table 1:** Comprehensive summary of therapeutic evidence and essential quality control (QC) parameters for exosome-based immunotherapy in colorectal cancer.

Study type	Exosome source/platform	Engineering/intervention strategy	Key findings/therapeutic impact	Ref.
Preclinical Studies	Mouse CRC cell lines	Neoantigen-painted exosomes combined with anti-PD-1 therapy	Elicited complete tumor regression and established durable immunological memory; synergized with checkpoint blockade.	(65)
	Mesenchymal stromal/stem cell-derived exosomes	Loaded with miR-125b-5p (targeting TNFR2 and FoxP3) + anti-PD-1	Significantly inhibited Treg function and enhanced anti-PD-1 efficacy, achieving ~80% tumor regression in CRC models.	(71)
	Primary CRC tumor-derived exosomes	Loaded with siRNAs targeting CCDC80 + immune checkpoint blockade	Suppressed CRC liver metastasis and chemoresistance; sensitized tumors to chemotherapy and enhanced antitumor immunity.	(45)
	Irradiated cancer cell-derived exosomes	Radiation-induced Immunogenic Cell Death (ICD)	Transferred IFN-stimulatory dsDNA to DCs, activating the cGAS/STING pathway and massively enhancing antitumor immunity.	(62)
	Tumor-derived exosomes (TEXs)	Curvature-sensing peptides disrupting TEX membranes + anti-PD-1	Reduced circulating exosomal PD-L1 levels, restored CD8+ T cell function, and enhanced anti-PD-1 efficacy.	(56)
	Tumor cell-derived nanovaccines	ER stress induction to upregulate HSP70 expression in secreted exosomes	Enhanced DC targeting and activation; produced robust antitumor immune responses and long-term immune memory.	(58)
Clinical Trials	Autologous ascites-derived exosomes (Phase I Trial)	Native exosomes administered in combination with GM-CSF	Demonstrated safety and feasibility; induced tumor-specific CTL responses, though overall clinical efficacy remained limited.	(23)
	Dendritic cell-derived exosomes (DEXs) (Early Clinical Trials)	DEXs loaded with tumor-associated antigens	Circumvented native TEX immunosuppression; demonstrated strong safety profiles and promising immunological responses.	(66, 69)
Translational Quality Control (QC) & Manufacturing	Physical Characterization	Size distribution (30–150 nm) via Nanoparticle Tracking Analysis (NTA); Morphology via Cryo-EM	Verifies structural integrity and uniformity; differentiates TEXs from apoptotic bodies.	(22, 72)
	Biochemical Purity	Enrichment of transmembrane (CD9, CD63) and cytosolic markers (TSG101); Absence of Calnexin	Validates endosomal origin; confirms removal of cellular debris and non-vesicular proteins.	(72, 73)
	Functional Potency & Cargo	T-cell activation assays; Quantification of target antigens and residual surface PD-L1	Ensures consistent dosing stoichiometry and verifies neutralization of immunosuppressive traits.	(22, 73)

Surface modification of exosomes represents a key engineering strategy to enhance their targeting specificity and immunostimulatory properties. Genetic engineering of parental cells to express targeting ligands on exosome surfaces enables directed delivery to specific immune cell populations or tumor sites (19, 20). For example, exosomes engineered to express anti-CD3 or anti-EGFR antibodies can specifically target T cells or tumor cells, respectively, enhancing the precision of immunotherapy (74, 75). Similarly, exosomes decorated with DC-targeting molecules, such as DEC-205 or DC-SIGN ligands, can improve antigen delivery to DCs and enhance T cell priming (68).

Cargo loading strategies allow for the incorporation of therapeutic molecules into exosomes to augment their immunotherapeutic effects. Recently, breakthrough advances in pharmaceutics and exosome engineering have further optimized these drug delivery systems, demonstrating that advanced loading techniques can significantly enhance the stability, precise targeting, and therapeutic payload capacity of exosomes. Although recently validated in other severe disease models (such as glioblastoma and stroke), these state-of-the-art delivery systems provide a robust technological framework that can be directly adapted for CRC immunotherapy (76–78). For instance, exosomes can be loaded with immunostimulatory cytokines, such as IL-2, IL-12, or IFN-γ, to enhance T cell activation and proliferation within the TME (19, 70). Loading exosomes with TLR agonists, such as CpG-ODN or poly(I:C), can activate innate immune pathways and promote DC maturation, thereby enhancing adaptive antitumor immunity (60, 61). Additionally, exosomes can be loaded with siRNAs or miRNAs to silence immunosuppressive genes in target cells, such as PD-L1 or TGF-β, reversing the immunosuppressive TME (45, 54).

The CRISPR/Cas9 system has been successfully delivered via engineered exosomes for genome editing applications in cancer immunotherapy (1, 20). Exosomes loaded with CRISPR components targeting immune checkpoint genes or immunosuppressive factors can induce permanent genetic modifications in tumor cells or immune cells, potentially achieving sustained therapeutic effects. This approach holds promise for reprogramming the TME at the genetic level to enhance immunotherapy responses.

Combination therapies involving engineered exosomes and immune checkpoint blockade have shown particular promise in preclinical CRC models. Exosomes engineered to deliver miR-125b-5p, which targets TNFR2 and FoxP3 to inhibit Treg function, significantly enhanced anti-PD-1 therapy efficacy in a mouse colon cancer model, achieving tumor regression in approximately 80% of treated mice compared to 30% with anti-PD-1 alone (71). Similarly, exosomes loaded with siRNAs targeting CCDC80, a gene associated with CRC liver metastasis and chemoresistance, sensitized tumors to chemotherapy and enhanced antitumor immunity when combined with checkpoint blockade (45).

Engineering exosomes to deliver tumor antigens and adjuvants simultaneously can create potent cancer vaccines that overcome the limitations of native TEXs. Biologically self-assembled tumor cell-derived nanovaccines, created by inducing endoplasmic reticulum (ER) stress in tumor cells to upregulate HSP70 expression in secreted exosomes, demonstrated enhanced DC targeting and activation, leading to robust antitumor immune responses and long-term immune memory (58). This approach leverages the natural exosome biogenesis machinery to produce vaccines with optimal antigen and adjuvant content without complex *in vitro* engineering.

The combination of exosome-based therapies with other immunomodulatory treatments offers additional opportunities for enhancing immunotherapy responses in cold CRC. Photodynamic therapy can be used to boost the release of immunogenic TEXs from tumor cells, creating an *in situ* vaccination effect (79). Exosome inhibitors that block TEX secretion or uptake can be combined with checkpoint blockade to simultaneously reduce immunosuppression and enhance T cell activity (55, 56). Curvature-sensing peptides that disrupt TEX membranes have been shown to reduce circulating exosomal PD-L1 levels and restore CD8+ T cell function, enhancing the efficacy of anti-PD-1 therapy in mouse models (56).

To further augment the clinical applicability of TEX-based therapies, nanomaterial-based approaches have been introduced as complementary strategies. Hybrid TEX-nanoparticle systems combine the natural tumor-homing ability and antigenic richness of TEXs with the high drug-loading capacity and controlled release properties of synthetic nanomaterials (80, 81). These exo-nanomaterials can be designed to respond to TME stimuli, such as low pH or hypoxia, enabling spatiotemporally controlled drug release at tumor sites (80). Integrating TEXs with smart synthetic delivery systems holds significant promise for enhancing the precision of CRC immunotherapy.

While TEXs remain the central focus of this review due to their unparalleled tumor antigen repertoire, it is important to briefly contextualize them against alternative, immune cell-derived exosome platforms, which serve as crucial comparative benchmarks and potential complementary therapies. For instance, unlike native TEXs which carry suppressive cargo, engineered DC-derived exosomes inherently express high levels of costimulatory molecules that can directly activate T cells (68, 75). Furthermore, NK cell-derived exosomes carrying cytotoxic molecules (75, 82), or macrophage-derived exosomes engineered to repolarize M2 TAMs (42, 82), represent parallel strategies. Ultimately, combining the broad antigen-presenting capabilities of engineered TEXs with the direct immune-activating properties of these complementary immune-derived vesicles could formulate the next generation of synergistic immunotherapies for cold CRC.

Despite the promise of engineered exosomes, several challenges must be addressed for clinical translation. Standardization of exosome isolation, characterization, and engineering methods is essential for reproducible therapeutic outcomes (22, 72). Scalable production of engineered exosomes with consistent quality remains a technical hurdle, particularly for clinical applications requiring large quantities (19, 21). Additionally, the long-term safety of engineered exosomes, particularly those containing genetic materials or synthetic components, requires thorough evaluation in preclinical models and clinical trials (20, 70).

## 5 Clinical translation and challenges

The translation of TEX-based immunotherapies from preclinical models to clinical applications faces several highly specific biological, technical, and regulatory challenges that must be addressed to realize their therapeutic potential for cold CRC. One of the primary obstacles is the heterogeneity of exosomes and the lack of standardized methods for their isolation and characterization (22, 72). The establishment of standardized protocols, adhering to the MISEV guidelines, is essential for clinical advancement, as detailed in the quality control section of **Table 1**. Furthermore, transitioning to Good Manufacturing Practice (GMP)-scale production exposes severe biomanufacturing bottlenecks. While scalable techniques like tangential flow filtration (TFF) are preferred for upscaling, they frequently suffer from membrane clogging due to protein aggregates and the highly mucinous nature of certain CRC subtypes (21, 72). Moreover, maintaining stringent batch-to-batch consistency—particularly regarding the density of MHC complexes and the stoichiometry of loaded adjuvants—remains a profound technical hurdle for commercial translation (19, 80). Beyond manufacturing, the selection of antigen sources presents a fundamental translational dilemma specific to CRC vaccine development. Utilizing autologous CRC-derived exosomes guarantees the inclusion of patient-specific neoantigens necessary to break immune tolerance in MSS tumors; however, it is logistically cumbersome and often yields insufficient exosome quantities from primary surgical resections (15, 65). Conversely, off-the-shelf allogeneic TEXs derived from established CRC cell lines offer highly scalable production but face the dual risks of insufficient personalized antigenic coverage and potential allogeneic MHC mismatch, which can induce rapid immune clearance rather than robust therapeutic cross-priming (61, 65). *In vivo* biodistribution also presents a pharmacological challenge. Systemically administered exosomes are rapidly cleared by the liver, spleen, and lungs, limiting their accumulation at tumor sites or draining lymph nodes (73, 83). While surface modification can improve targeting, this may simultaneously alter exosome clearance and invoke neutralizing immunogenicity, particularly when utilizing synthetic components (19–21, 72). Finally, the clinical deployment of engineered TEX vaccines is stymied by profound regulatory classification uncertainties. It remains ambiguous across global regulatory frameworks whether artificially loaded or CRISPR-edited TEXs should be classified as conventional biological products (akin to standard vaccines) or stringently regulated as advanced therapy medicinal products (ATMPs). Clear regulatory guidelines defining specific potency assays and safety evaluations for these hybrid nano-biologicals are urgently needed to facilitate their eventual marketing approval (73, 83).

## 6 Future perspectives

Rather than reiterating the well-established immunosuppressive mechanisms of native TEXs, the future of this field must be strictly forward-looking, focusing on precision engineering and rational combinatorial therapies. A critical next step is the advancement of personalized, neoantigen-based TEX vaccines. Leveraging patient-specific neoantigens loaded into engineered TEXs could elicit highly targeted CTL responses, providing a personalized approach to overcoming the diverse mutational landscape of CRC (15, 65).

Furthermore, the synergistic potential of rational TEX-ICI combinations will define the next phase of clinical trials. Because engineered TEXs can robustly prime T cells while ICIs release the brakes on T cell exhaustion, integrating these platforms with standard-of-care treatments—such as ICD-inducing radiotherapy or targeted CSF1R inhibitors—could create a highly permissive TME (5, 8, 10, 84).

Looking beyond current milestones, several proposed future applications remain in the exploratory phase but offer exciting opportunities. Although not yet established in clinical practice, single-cell and spatial transcriptomics are being utilized in basic research to provide high-resolution insights into TEX heterogeneity (1, 85). In the future, artificial intelligence and machine learning approaches could hypothetically be leveraged to predict exosomal cargo composition and optimize patient selection (73, 83). In terms of clinical maturity, the development of exosome-based biomarkers is currently transitioning from preclinical validation to early clinical observation. Evaluating circulating exosomal PD-L1 and specific miRNA profiles (86, 87) in large-scale prospective trials will be critical for facilitating patient stratification and dynamic response monitoring (30, 31, 88) (**Figure 2**).

**Figure 2:**
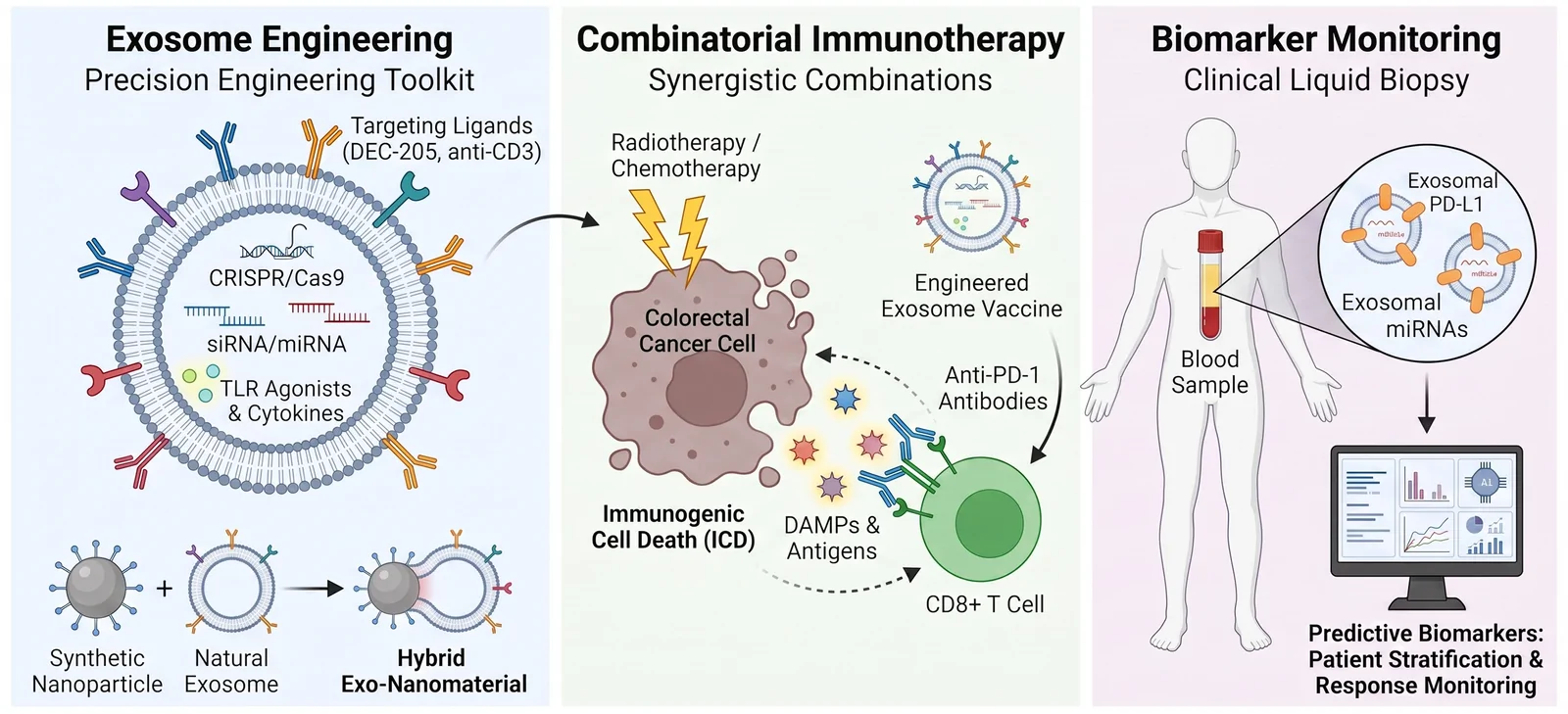
Advanced engineering strategies, combinatorial immunotherapies, and clinical translation of exosome-based therapeutics in colorectal cancer (CRC). (Left) The precision exosome engineering toolkit includes surface modification with specific targeting ligands (e.g., DEC-205 for dendritic cells or anti-CD3 for T cells) and the internal loading of therapeutic cargos, such as CRISPR/Cas9 systems, siRNA/miRNAs, Toll-like receptor (TLR) agonists, and cytokines. Additionally, natural exosomes can be fused with synthetic nanoparticles to generate hybrid exo-nanomaterials with enhanced delivery capabilities. (Middle) Synergistic combinatorial immunotherapy leverages standard treatments (radiotherapy or chemotherapy) to trigger immunogenic cell death (ICD) and release damage-associated molecular patterns (DAMPs) alongside tumor antigens. Integrating engineered exosome vaccines with immune checkpoint inhibitors (anti-PD-1 antibodies) vigorously primes CD8+ T cells to eradicate CRC cells. (Right) In clinical liquid biopsy, circulating exosomal PD-L1 and miRNAs derived from patient blood samples serve as predictive biomarkers for precise patient stratification, real-time response monitoring, and AI-assisted clinical decision-making.

## 7 Conclusion

Current preclinical evidence demonstrates that tumor-derived exosomes (TEXs) act as a highly paradoxical double-edged sword in colorectal cancer: while native TEXs actively orchestrate the immunosuppressive “cold” tumor microenvironment, engineered TEXs can be successfully repurposed into potent, antigen-rich vaccine platforms. However, broad clinical translation is currently prevented by significant developmental bottlenecks, primarily the lack of standardized isolation protocols, the logistical challenges of scalable manufacturing, and the complex necessity of neutralizing their native immunosuppressive cargo safely. Therefore, the most important next step for the field is the rigorous clinical evaluation of rational combinatorial therapies—specifically, integrating personalized, neoantigen-engineered TEX vaccines with immune checkpoint inhibitors—to definitively reprogram cold CRC into an immune-responsive state in human trials.
